# Medical News and Miscellany

**Published:** 1889-08

**Authors:** 


### MEDICAL NEWS AND MISCELLANY.

---

**Death of Mrs. Gibson.**—We regret to chronicle the death of Mrs. M. E. Gaillard's mother, Mrs. Ellen Gibson, relict of the late Prof. Charles Bell Gibson, of Richmond, Va. Mrs. Gibson died on the 8th July ult., in New York, at the residence of her daughter.

---

**Dr. J. D. Osborn,** of Cleburne, was recently, at the annual convention of the Grand Lodge of Knights of Honor, held at Tyler, re-elected State Medical Examiner. This is the third or fourth consecutive re-election of Dr. Osborn to this honorable and responsible position, and is a most flattering testimonial to his worth and fidelity, as well as to his ability as a medical man. We congratulate the doctor, and also congratulate the order upon the possession of so faithful a Supreme Examiner.

---

**Important Announcement.**—Dr. H. C. Ghent withdraws his endorsement of "The Biography of Contemporary Physicians of Texas," on personal grounds.

In resuming the canvass for this work, we published the endorsement of some of our early subscribers—among them, the enthusiastic one of Dr. H. C. Ghent. Dr. H. C. Ghent notifies Dr. Tobin, the President of the Biographical Publishing company, that he withdraws it, Dr. Daniel being no longer a friend of his, he says, he does not think, as he did in '84, that "he is very man to write it;" and fears that Dr. Daniel will not do him justice. We make this announcement in justice to Dr. H. C. Ghent.

F. E. DANIEL.

---

**Complete your Record.**—Time and again the Secretary has called on members of the Texas State Medical Association, to furnish him the data to complete their record on the roll of members. What is wanted is to know when and where each member graduated and when joined the association, as well as age at time of joining, and of what state a native. Members may think this is of little importance; it is very important, not only as a part of the medical history of the association, but the secretary is often interrogated by the Chief Surgeon or Medical Director of large Life Insurance Companies on these points; and ones appointment, at any time, as local, district, a State Medical Examiner for some large Insurance Company with a fee of $5 to $20 for each examination *may depend on their record*, or the absence of it.

A word to the wise ought to be sufficient.

---

**To the Physicians of Texas.**—We wish to say, that the Biography of Contemporary Physicians of Texas, now being compiled and rapidly prosecuted by the editor of this journal, will not consist of a dissertation on the life and character of those who subscribe to it; there will be no eulogies, no fulsome praise, no discriminations and comparisons, no comments, no expressions of opinion by the author; but will consist for the most part, of a brief chronological record of the more important events in the life of each; of facts, briefly (but fully) stated, and those facts *are to be furnished by subscribers themselves.* They will be properly arranged, and where necessary, elaborated, *edited* by the editor, and put in good shape for publication. Of course some will be longer than others, but in the main, all will be as short as is consistent, with a clear statement of fact. Moreover, in order that each subscriber, and those who contemplate subscribing may "know just what it is proposed to say" about each, proof sheets will be submitted to each, of whatever is prepared for publication, from the data furnished by subscribers, for their approval, alteration or correction. Also where desired, a print from the engraving made from photo, will be submitted before being published. In this way it is hoped and expected to avoid any mistakes, and friend or foe, the editor could not possibly do any thing but justice to each subscriber.

We beg to call attention to the advertisement of the *Manhattan Life Insurance Company*, of New York. This is one of the strongest and most popular companies extant; and as life insurance has become a necessity, our readers will do well to investigate the claim of the Manhattan before placing their application.

